# The Association of Acute Signs and Symptoms of COVID-19 and Exacerbation of Depression and Anxiety in Patients With Clinically Mild COVID-19: Retrospective Observational Study

**DOI:** 10.2196/43003

**Published:** 2023-01-30

**Authors:** Sumi Sung, Su Hwan Kim, Changwoo Lee, Youlim Kim, Ye Seul Bae, Eui Kyu Chie

**Affiliations:** 1 Office of Hospital Information Seoul National University Hospital Seoul Republic of Korea; 2 Biomedical Research Institute Seoul National University Hospital Seoul Republic of Korea; 3 Department of Family Medicine Seoul National University Hospital Seoul Republic of Korea; 4 Department of Radiation Oncology College of Medicine Seoul National University Seoul Republic of Korea; 5 Institute of Radiation Medicine Medical Research Center Seoul National University Seoul Republic of Korea

**Keywords:** COVID-19, depression, anxiety, vital signs, symptoms, electronic health records

## Abstract

**Background:**

To date, the association between acute signs and symptoms of COVID-19 and the exacerbation of depression and anxiety in patients with clinically mild COVID-19 has not been evaluated.

**Objective:**

This study was designed to assess the correlation between acute signs and symptoms of COVID-19 and the exacerbation of depression and anxiety in patients with clinically mild COVID-19 at a residential treatment center in South Korea.

**Methods:**

This retrospective study assessed 2671 patients with COVID-19 admitted to 4 residential treatment centers operated by Seoul National University Hospital, South Korea, from March 2020 to April 2022. Depression and anxiety were assessed using the 2-item Patient Health Questionnaire (PHQ-2) and 2-item Generalized Anxiety Disorder (GAD-2) scale, respectively. The exacerbation of depression and anxiety symptoms was identified from the differences in PHQ-2 and GAD-2 scores between admission and discharge, respectively. The patients’ clinical characteristics, including acute signs and symptoms of COVID-19, GAD-2 and PHQ-2 scores, were obtained from electronic health records. Demographic characteristics, a summary of vital signs, and COVID-19 symptoms were analyzed and compared between the patient groups with and those without exacerbated PHQ-2 and GAD-2 scores using the chi-square test. We applied logistic regression to identify the association between acute signs and symptoms of COVID-19 and the exacerbation of depression and anxiety.

**Results:**

Sleep disorders were associated with exacerbated depression (odds ratio [OR] 1.09, 95% CI 1.05-1.13) and anxiety (OR 1.1, 95% CI 1.06-1.14), and the sore throat symptom was associated with exacerbated anxiety symptoms (OR 1.03, 95% CI 1.00-1.07). Patients with abnormal oxygen saturation during quarantine were more likely to have exacerbated depression (OR 1.27, 95% CI 1.00-1.62), and those with an abnormal body temperature during quarantine were more likely to experience anxiety (OR 1.08, 95% CI 1.01-1.16). As anticipated, patients who experienced psychological symptoms at admission were more likely to experience depression (OR 1.91, 95% CI 1.52-2.41) and anxiety (OR 1.98, 95% CI 1.54-2.53). Meanwhile, the PHQ-2 and GAD-2 scores measured at admission revealed that lower the score, higher the possibility of exacerbation of both depression (OR 0.15, 95% CI 0.11-0.22) and anxiety (OR 0.13, 95% CI 0.10-0.19).

**Conclusions:**

Results from this study suggest the importance of further interventions for patients with abnormal oxygen saturation, abnormal body temperatures, sore throat, and sleep disorder symptoms or initial psychological symptoms to mitigate the exacerbation of depression and anxiety. In addition, this study highlights the usability of short and efficient scales such as the PHQ-2 and GAD-2 in the assessment of the mental health of patients with clinically mild COVID-19 symptoms who were quarantined at home during the pandemic era.

## Introduction

The ongoing COVID-19 pandemic has not only affected physical health but also induced mental health issues such as depression, anxiety, stress, or insomnia [1-3]. A systematic review on the prevalence of psychological symptoms in the general population during the COVID-19 pandemic reported that the prevalence of depressive symptoms ranged from 14.6% to 48.3% and that of anxiety ranged from 6.33% to 50.9%, which is more than double the prevalence in the nonpandemic era [4]. South Korea is no exception because of its increased prevalence of psychological symptoms associated with depression and stress [5-7]. These remarkable increases in psychological symptoms are known to contribute to mental health problems such as anxiety, depression, insomnia, somatization, posttraumatic stress disorder, self-harm, and suicidal thoughts and behaviors. Thus, the importance of early detection and management of mental health disorders cannot be overemphasized [6,8].

Patients confirmed with a COVID-19 diagnosis most commonly experience physical symptoms such as fever, cough, and fatigue [9,10]. In particular, nonhospitalized patients with a clinically mild COVID-19 infection have fatigue, headache, and sneezing as the most common acute symptoms, and fatigue and reduced smell and taste as the most severe symptoms [11]. While transitioning to focusing on the post–COVID-19 condition, symptoms such as fatigue may persist as long as 12 weeks in the long-term phase, and these symptoms are known to affect patients’ mental health and the quality of life [11,12].

Despite attempts to explore acute and persistent symptoms of COVID-19 and patients’ mental health affected by persistent symptoms, little is known about the association between physical and psychological symptoms of acute COVID-19. Ismael et al [13] found that an increased number of COVID-19 symptoms may be associated with depression, anxiety, and posttraumatic symptoms. Perlis et al [14] reported that patients with COVID-19 who reported headaches during acute infection appeared to have an elevated risk of depression. During the transition to post–COVID-19 condition [15], it is necessary to examine the occurrence of acute signs and symptoms, changes in mental health, and their association while community-dwelling confirmed patients were quarantined.

The Korean government has operated a residential treatment center (RTC) in response to the COVID-19 pandemic [16]. In the early stages of the COVID-19 outbreak, patients with COVID-19 could not be admitted to the hospital because of a lack of negative-pressure isolation beds. Accordingly, the government admitted all confirmed patients with clinically mild conditions to RTCs and transferred them to the hospital if their medical condition deteriorated to require admission-based care. Since then, with the rapid increase in the number of patients with COVID-19, high-risk patient groups with mild conditions have been selectively isolated to RTCs because it was difficult to isolate all confirmed patients at RTCs.

As patients with confirmed COVID-19 without symptoms and those with mild symptoms were being monitored in quarantine at home, it was not easy to explore the status of their acute signs and symptoms and psychological diseases such as depression and anxiety. Therefore, it is important to ascertain the association between COVID-19–related acute signs and symptoms, and depression and anxiety, based on the accumulated retrospective data. With this background, we analyzed the association between acute signs and symptoms of COVID-19 and exacerbation of depression and anxiety in nonhospitalized patients with clinically mild COVID-19 symptoms who were admitted to RTCs.

## Methods

### Study Design

This retrospective study was performed at Seoul National University Hospital (SNUH).

### Study Setting

The study setting was 4 RTCs operated by the SNUH in accordance with government guidelines [17]. The government RTC operating guidelines were periodically updated in consideration of the number of newly confirmed cases at the time the RTC operation and distribution of confirmed cases at the national level. The confirmed patients were assigned to each RTC by the government in accordance with this guideline. During the early phase of the pandemic, from December 2020 to November 2021, following the relevant Korean guidelines, all clinically mild cases among the confirmed cases in South Korea were quarantined at the RTC. With a rapid increase in the number of confirmed cases, all quarantine policies were modified to prioritize those who are more vulnerable to deterioration of their COVID-19 infection. Selection criteria for RTC admission include unvaccinated older individuals and patients in residential environments vulnerable to the spread of infection. All patients were quarantined independently in a separate room. All costs related to quarantine at an RTC were fully covered by the government. Quarantined patients requiring oxygen supply due to a persistent saturation of percutaneous oxygen (SpO_2_) of <94%, or those with a clinical condition requiring medical intervention, were transferred to the hospital. Table 1 shows the location, size in terms of the number of beds, operation period, and accumulated confirmed COVID-19 cases at the national level for operated RTCs in detail.

**Table 1:** Details of residential treatment centers operated by the Seoul National University Hospital.

Name	Location	Beds, n	Operation period (accumulated confirmed COVID-19 cases at the national level during the operational period, n) [17]
A	Near Daegu city in North Gyeongsang province	99	March 2020 to April 2020 (7834)
B	Nowon-gu, Seoul	124	August 2020 to October 2020 (12,206)
C	Seongnam city in Gyeonggi province	225	August 2020 to September 2020 (9506)
D	Seongnam city in Gyeonggi province	334	December 2020 to January 2021 (43,998) July 2021 to April 2022 (17,079,907)

### Study Participants

Adult patients with a confirmed COVID-19 diagnosis admitted to 4 RTCs operated by the SNUH from March 2020 to April 2022 were included in this study. Patients who tested positive for COVID-19 on the reverse transcriptase–polymerase chain reaction test but had clinically mild conditions were subjected to RTC admission. A total of 5199 patients were admitted during the study period. Patients with missing data were excluded from the analysis.

### Data Sources

Using the SNUH information and communication technology–based remote patient monitoring (RPM) system [18], it was mandatory for every patient admitted to the RTC operated by the SNUH to use the mobile app and to self-report their past medical history, vital signs, subjective acute COVID-19 symptoms, and psychological symptoms. Reporting was done once on admission, twice a day during the quarantine period, and once on discharge. These data were automatically stored in the hospital information system at the SNUH. The records were monitored in real time by health care providers. For any missing data in the records, health care providers directly checked the patient and filled in the missing data. We finally extracted these electronic health records using the SNUH Patient Research Environment (also known as “SUPREME”)—a clinical data warehouse at the SNUH. The data sources in the final analysis included RTC admission notes, daily progress notes, and discharge notes in the electronic health record.

Data on patients’ depressive and anxiety symptoms were extracted from the RTC admission and discharge notes. Patients’ self-reported scores on the 2-item Patient Health Questionnaire (PHQ-2)—a subscale of the Patient Health Questionnaire 9 [19]—and 2-item Generalized Anxiety Disorder Scale (GAD-2)—a subscale of the Generalized Anxiety Disorder Scale (GAD-7) [20]—via a mobile app at admission and discharge. One of the PHQ-2 items was, “Over the last 2 weeks, how often have you been bothered by any of the following problems?” with response options of “Little interest or pleasure in doing things,” and “Feeling down, depressed, or hopeless” on a 4-point Likert scale. Scores ranging from 0 to 6, with a cutoff score of ≥3 indicating depressive symptoms. The PHQ-2 was reported to have a sensitivity of 0.79 and a specificity of 0.86 for any depressive disorders [21]. One of the GAD-2 items was, “Over the last 2 weeks, how often have you been bothered by any of the following problems?” with response options of “Feeling nervous, anxious, or on the edge” and “not being able to stop or control worrying” with a 4-point Likert scale. Scores range from 0 to 6, with a cutoff score of ≥3 indicating anxiety symptoms. The GAD-2 was reported to have a sensitivity of 0.65 and a specificity of 0.88 as a general screening tool for anxiety disorders [22].

Patient demographic characteristics (eg, age, sex, and admitted RTC site), medical history (diabetes mellitus, hypertension, cardiovascular disease, respiratory disease, sleep disorder, and psychological treatment), and initial psychological symptoms such as depression and panic were extracted from the RTCs’ admission notes. Patients’ smoking statuses were extracted from the RTCs’ discharge notes.

Patients’ self-measured vital signs and acute COVID-19 symptoms were extracted from the RTCs’ daily progress notes, which were collected via a mobile app at least twice a day. The vital signs included systolic blood pressure, diastolic blood pressure, heart rate, respiratory rate, body temperature (BT), and SpO_2_. Acute COVID-19 symptoms consisted of the presence or absence of respiratory and nonrespiratory symptoms. Respiratory symptoms included cough, sputum production, fever, rhinorrhea, sore throat, dyspnea, and chest pain. Nonrespiratory symptoms include nausea, vomiting, abdominal discomfort, pain, constipation, diarrhea, abdominal pain, and sleep disorders.

The loss of taste and smell was extracted from the RTCs’ discharge notes. At discharge, participants reported the presence or loss of smell and taste.

### Statistical Analysis

Data preparation was conducted before data analysis. The exacerbation of depressive symptoms’ and anxiety symptoms’ cases were identified from the differences between PHQ-2 scores and GAD-2 scores at admission and discharge, respectively. Criteria for abnormal vital signs, in consultation with an infection medicine specialist, are as follows: a systolic blood pressure above 140 mm Hg or below 60 mm Hg, a diastolic blood pressure above 90 mm Hg or below 30 mm Hg, a heart rate above 110 bpm or below 40 bpm, respiratory rate above 21/minute or below 8/minute, BT above 38.0 °C or below 35.0 °C, and an SpO_2_ below 94%. The number of days with each abnormal vital sign and acute COVID-19 symptom was calculated.

Demographic characteristics, summary of vital signs, and COVID-19 symptoms were analyzed and compared between the patient groups with and those without exacerbated PHQ-2 and GAD-2 scores, using the chi-square test. We then applied logistic regression with exacerbation of depressive and anxiety symptoms as dependent variables, and sociodemographic features, past medical history, initial psychological symptoms, initial PHQ-2 or GAD-2 scores, average number of days with abnormal vital signs, and average number of days with acute COVID-19 symptoms as independent variables, using R statistical software (version 4.2; R Project for Statistical Computing). Statistical significance was tested at α=.01.

### Ethical Considerations

This study was approved by the SNUH’s institutional review board (H-2105-158-1221) and was conducted in accordance with the relevant guidelines and regulations. Informed consent was waived by the institutional review board considering the study design and adherence to the relevant guidelines. In particular, study data were deidentified to protect privacy and preserve the confidentiality of the study participants.

## Results

### Patients’ Clinical Characteristics

In total, 2671 individuals reported COVID-19 infection without any missing values. The clinical characteristics of the patients are presented in Table 2. The mean age was 41.1 (SD 14.1) years, and the mean quarantine period was 7.07 days. Overall, 1242 (46.5%) participants were female, and 44 (1.6%) were admitted at RTC A, 324 (11.8%) at RTC B, 819 (30.7%) at RTC C, and 1494 (55.9%) at RTC D. A total of 746 (27.9%) patients were infected by the alpha variant of SARS-CoV-2, 1563 (58.5%) by the delta variant, and 362 (13.6%) by the omicron variant. Overall, 141 (5.3%) participants had a past medical history of diabetes mellitus, 361 (13.5%) had a medical history of hypertension, 168 (6.3%) had a medical history of sleep disorders, 158 (5.9%) had a history of psychological treatment. Upon admission, 1273 (47.7%) participants had initial psychological symptoms. Detailed clinical characteristics upon exacerbation of PHQ-2 and GAD-2 scores are presented in Multimedia Appendix 1.

**Table 2:** Patients’ clinical characteristics (N=2671).

Clinical characteristics			Participants, n (%)
**Demographic data**			
	Females	1242 (46.5)	
	Aged under 30 years	748 (28.0)	
	Aged 30 to 39 years	500 (18.7)	
	Ages 40 to 49 years	545 (20.4)	
	Aged 50 to 59 years	556 (20.8)	
	Aged 60 years or older	322 (12.1)	
	Admitted to residential treatment center A	44 (1.6)	
	Admitted to residential treatment center B	314 (11.8)	
	Admitted to residential treatment center C	819 (30.7)	
	Admitted to residential treatment center D	1494 (55.9)	
	Infected with the SARS-CoV-2 alpha variant	746 (27.9)	
	Infected with the SARS-CoV-2 delta variant	1563 (58.5)	
	Infected with the SARS-CoV-2 omicron variant	362 (13.6)	
**Medical history**			
	Diabetes mellitus	141 (5.3)	
	Hypertension	361 (13.5)	
	Cardiovascular disease	81 (3.0)	
	Respiratory disease	44 (1.6)	
	Sleep disorder	168 (6.3)	
	Psychological treatment	158 (5.9)	
Initial psychological symptoms			1273 (47.7)
**Initial score on the 2-item Patient Health Questionnaire**			
	0	1641 (61.4)	
	1	367 (13.7)	
	2 or above	663 (24.8)	
**Initial score on the 2-item Generalized Anxiety Disorder Scale**			
	0	1461 (54.7)	
	1	354 (13.3)	
	2 or above	856 (32.0)	
**Smoking status**			
	Never smoked	1600 (59.9)	
	Ex-smoker	565 (21.2)	
	Smoker	506 (18.9)	
**BMI category**			
	Underweight or normal	1182 (44.3)	
	Overweight	580 (21.7)	
	Obese	909 (34.0)	
**Abnormal vital signs**			
	Systolic blood pressure	564 (21.1)	
	Diastolic blood pressure	1073 (40.2)	
	Heart rate	86 (3.2)	
	Respiratory rate	730 (27.3)	
	Body temperature	375 (14.0)	
	Saturation of percutaneous oxygen	72 (2.7)	
**Acute respiratory COVID-19–related symptoms**			
	Cough	1759 (65.9)	
	Sputum	1543 (57.8)	
	Fever	544 (20.4)	
	Rhinorrhea	1276 (47.8)	
	Sore throat	1153 (43.2)	
	Dyspnea	250 (9.4)	
	Chest pain	421 (15.8)	
**Acute nonrespiratory COVID-19–related symptoms**			
	Nausea	207 (7.7)	
	Vomit	83 (3.1)	
	Abdominal discomfort	453 (17.0)	
	Pain	1047 (39.2)	
	Constipation	426 (15.9)	
	Diarrhea	490 (18.3)	
	Abdominal pain	186 (7.0)	
	Sleep disorder	624 (23.4)	
	Loss of smell	1264 (47.3)	
	Loss of taste	1243 (46.5)	

### PHQ-2 and GAD-2 Scores Measured at Admission and Discharge

**Figure 1:**
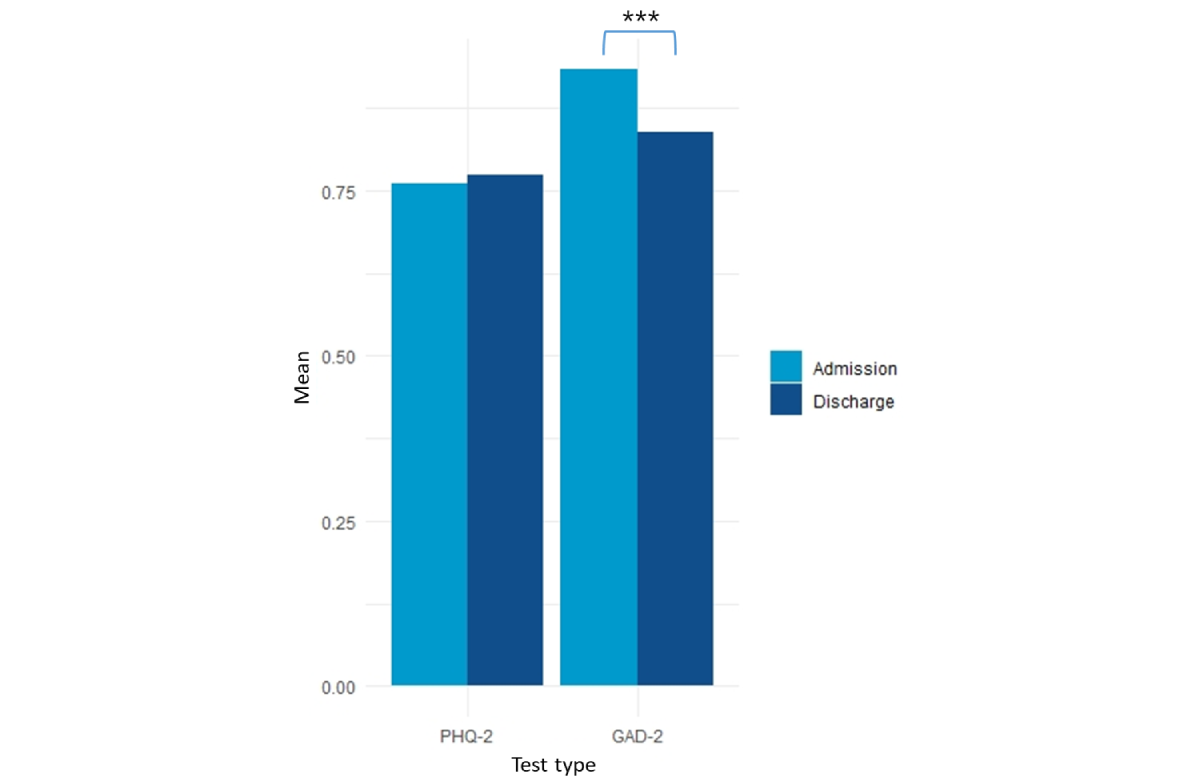
Scores on the 2-item Patient Health Questionnaire (PHQ-2) and the 2-item Generalized Anxiety Disorder Scale (GAD-2) measured at admission and discharge.

The PHQ-2 and GAD-2 scores measured at admission and discharge are presented in Figure 1. At admission, the mean PHQ-2 score was 0.76 (SD 1.17). Of a total of 2671 patients, 663 (24.8%) had a PHQ-2 score of 2 or higher. The mean GAD-2 score was 0.93 (SD 1.27), and 856 (32.0%) had a score of 2 or higher. At discharge, the mean PHQ-2 score was 0.77 (SD 1.18), and 675 (25.3%) had a score of 2 or higher. The mean GAD-2 score was 0.84 (SD 1.21), and 744 (27.9%) had a score of 2 or higher.

**Figure 2:**
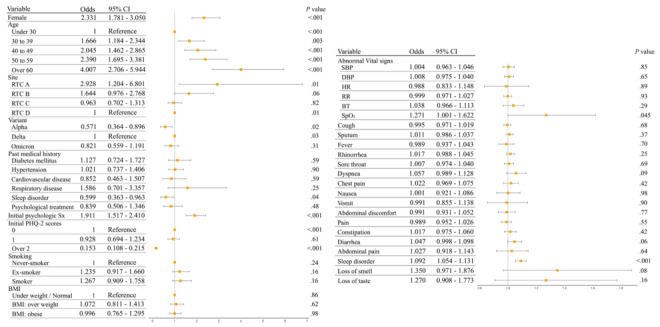
Forest plot showing the association of acute signs and symptoms of COVID-19 and exacerbation of depression. BT: body temperature; DBP: diastolic blood pressure; HR: heart rate; PHQ-2: 2-item Patient Health Questionnaire; RR: respiratory rate; RTC: residential treatment center; SBP: systolic blood pressure; SpO_2_: saturation of percutaneous oxygen.

A total of 535 patients showed exacerbation of depression. In fully adjusted models, an abnormal SpO_2_ was associated with increased odds of exacerbation of depression (odds ratio [OR] 1.27, 95% CI 1.00-1.62). Among acute COVID-19 symptoms, presence of sleep disorder was associated with exacerbation of depression (OR 1.09, 95% CI 1.05-1.13). Women demonstrated higher odds of exacerbation of depression than men (OR 2.33, 95% CI 1.78-3.05) and higher odds of exacerbation of depression with increasing age (>60 years; OR 4.01, 95% CI 2.71-5.93). Patients admitted at RTC A demonstrated higher odds of exacerbation of depression than those who were admitted at RTCs B, C, and D (OR 2.93, 95% CI 1.24-6.93), and those diagnosed with COVID-19 due to the alpha variant of SARS-CoV-2 showed lower odds of symptom exacerbation than those with the delta variant (OR 0.57, 95% CI 0.36-0.90). Past medical history of sleep disorders demonstrated lower odds to associate with exacerbation (OR 0.60, 95% CI 0.36-0.98), whereas presence of initial psychological symptoms was associated with exacerbation (OR 1.91, 95% CI 1.52-2.41). The higher patients’ PHQ-2 scores at admission demonstrated lower odds of exacerbation of depression (OR 0.15, 95% CI 0.11-0.22). Figure 2 illustrates the ORs from the regression models adjusted for the patients’ clinical characteristics.

### Association of Acute Signs and Symptoms of COVID-19 and Exacerbation of Anxiety

**Figure 3:**
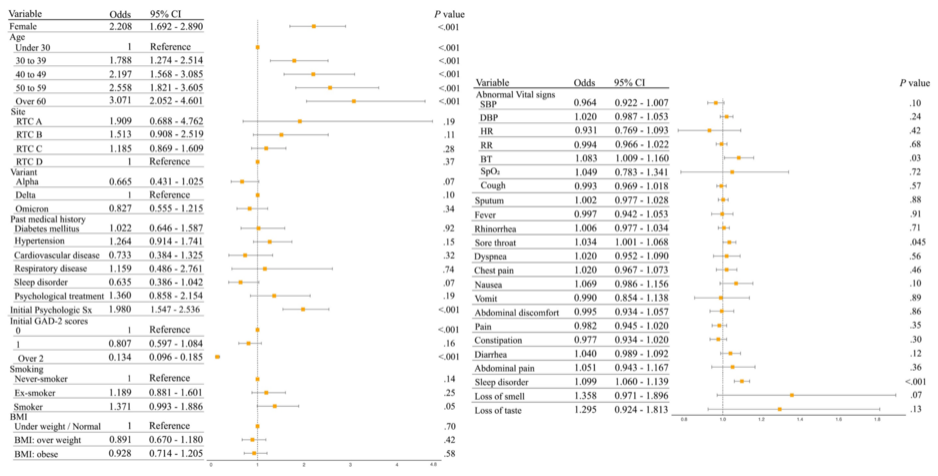
Association of acute signs and symptoms of COVID-19 and exacerbation of anxiety. BT: body temperature; DBP: diastolic blood pressure; HR: heart rate; PHQ-2: 2-item Patient Health Questionnaire; RR: respiratory rate; RTC: residential treatment center; SBP: systolic blood pressure; SpO_2_: saturation of percutaneous oxygen.

A total of 523 patients presented exacerbation of anxiety. Among abnormal vital signs, abnormal BT was associated with exacerbation of anxiety (OR 1.08, 95% CI 1.01-1.16). Among acute COVID-19 symptoms, the presence of sleep disorders and sore throat were associated with exacerbation of anxiety (sleep disorders: OR 1.1, 95% CI 1.06-1.14; sore throat: OR 1.03, 95% CI 1.00-1.07). Women showed higher odds of exacerbation of anxiety than men (OR 2.21, 95% CI 1.69-2.88) and higher odds of exacerbation of anxiety with increased age (>60 years; OR 3.07, 95% CI 2.05-4.60). Presence of initial psychological symptoms was associated with the exacerbation of anxiety (OR 1.98, 95% CI 1.54-2.53). The higher patients’ GAD-2 scores at admission indicated lower odds of exacerbation of anxiety (OR 0.13, 95% CI 0.10-0.19). Figure 3 illustrates the ORs from the regression models adjusted for patients’ clinical characteristics.

## Discussion

### Principal Findings

To the best of our knowledge, this study is the first to explore the association between acute signs and symptoms of COVID-19 and the exacerbation of depression and anxiety in patients with clinically mild COVID-19 [13-15]. Among sociodemographic features, we found that female sex and older age affected the likelihood of exacerbation of depression and anxiety in patients with clinically mild COVID-19, which is consistent with previous studies [23-26]. In addition, RTC A, located in Daegu and Gyeongbuk province, where patients in the early phase of COVID-19 with social stigma were admitted, was found to be highly associated with exacerbation of anxiety.

The most common acute COVID-19 symptoms are cough, sputum, rhinorrhea, sore throat, and loss of smell and taste. The proportion of these symptoms was ~40%-65%, which was relatively lower than that reported in previous studies on acute and persistent symptoms in nonhospitalized patients with COVID-19 [11]. This may reflect the results of triage, which incorporates multiple symptoms in accordance with the hospitalization policy of the Korea Disease Control and Prevention Agency. The types of acute COVID-19 symptoms most commonly complained of were consistent with those reported in previous studies in that fever, cough, myalgia, and fatigue were described as common symptoms of COVID-19 [9,10] and loss of smell and taste as prevalent and relatively discriminative symptoms of COVID-19 [27-29]. Sputum production has been regarded as a less common symptom of early COVID-19 [30,31] but was reported as one of the most common symptoms in this study. The reason for the high proportion of sputum production seems to be that study participants included patients infected with the omicron variant of SARS-CoV-2 whose complains of sputum symptoms were relatively high [32].

Among acute COVID-19 symptoms, patients who experienced sleep disorder symptoms more frequently during quarantine showed higher odds of exacerbation of depression (*P*<.001) and anxiety (*P*<.001) than those who did not. Sleep disorders are a core symptom of depression and anxiety, and this study shows a strong association between them, which is consistent with those reported in previous studies [33-35]. We also found that those who had experienced sore throat more frequently during quarantine had an elevated risk of anxiety symptoms (*P*<.05). The more the patient experiences sore throat, which is one of the representative upper respiratory symptoms of COVID-19 infection with the omicron variant, the higher the risk of exacerbation of anxiety, which means that early active intervention for mental health may be necessary. We expected that other similarly frequently reported acute symptoms such as cough, sputum production, rhinorrhea, and loss of smell and taste would also be associated with exacerbation of depression and anxiety, but this correlation was not found. Although it is difficult to generalize the results of this study, the association between physical symptoms and depression and anxiety is well known, and it is also well known that depression and anxiety accompany somatic symptoms. Thus, in the acute phase within 1 week of COVID-19 infection, health care providers should examine both physical and mental health during the treatment course of patients with acute COVID-19.

On the contrary, the vital signs of COVID-19 were stable, with a small proportion of patients presenting abnormal ranges during quarantine at the RTC. These results indicate that most patients admitted to the RTC had clinically mild conditions and did not require inpatient treatment. Patients who presented abnormal SpO_2_ during quarantine demonstrated higher odds of exacerbation of depression (*P*<.05) than those who did not. In addition, patients who had an abnormal BT during quarantine demonstrated higher odds of exacerbation of anxiety (*P*<.05). It is important to note that patients who often had unstable vital signs during quarantine showed the possibility of exacerbation to depression and anxiety due to exposure.

As anticipated, patients who had experienced psychological symptoms at admission had higher odds of exacerbation of depression (*P*<.001) and anxiety (*P*<.001) than those who did not. Meanwhile, the PHQ-2 and GAD-2 scores measured at admission showed that lower the score, higher the association between exacerbation of both depression (*P*<.001) and anxiety (*P*<.001). In addition, patients who had received previous psychological treatment were less likely to experience exacerbation of depression (*P*<.001). The group that received psychological treatment might have had a higher PHQ-2 or GAD-2 score at admission. Thus, these results can be interpreted as follows: higher the PHQ-2 or GAD-2 score at admission, narrower the range of scores that can be exacerbated.

The novelty of this study is the use of both contactless RPM systems and screening scales. First, data were obtained through a contactless RPM system for patients with COVID-19. More than 80% of patients with COVID-19 had clinically mild conditions, and these patients were quarantined at home or RTCs, not medical institutions, and monitored with a contactless RPM system. The establishment of a contactless RPM system for patients with clinically mild conditions who do not require intensive care unit care has been gradually attempted in various studies [36-38], and the SNUH has also established a contactless RPM system [18]. The data analyzed in this study were also the results of establishing the system at 4 RTCs operated by the SNUH.

Second, the results of this study provide supporting evidence that depression and anxiety can be assessed by the PHQ-2 and GAD-2, which are useful and efficient scales for patients with COVID-19 quarantined at home. As the prevalence of mood disorders has increased after the onset of the COVID-19 pandemic [4-7], concerns about individuals’ mental health are growing. Patients with COVID-19 who are self-isolated at home or at an RTC might be in a clinically mild condition and are not fully aware of the need for mental health assessment. Since they are not hospitalized and have limited access to medical personnel, it is necessary to assess the mental health of patients using a readily accessible simple convenient tool. In that sense, the PHQ-2 and GAD-2, which have already been validated as having high sensitivity and specificity as the PHQ-9 and GAD-7, may be regarded as useful screeners and efficient tools for monitoring treatment progress and outcomes in primary medical settings [21,39-43]. Furthermore, the results from this study suggest the importance of considering further interventions that might mitigate the exacerbation of depression and anxiety associated with acute signs and symptoms during the post–COVID-19 condition [15].

### Limitations

This study is not without its limitations. First, this was a retrospective observational study; therefore, causal relationships cannot be inferred, and further analysis of additional data such as socioeconomic factors, not collected in the hospital information system at the SNUH, was not possible. Second, the data analyzed were retrieved from a single tertiary hospital operating 4 RTCs of different sizes in accordance with government regulations. The possibility of influence or correlation with various unmeasured variables such as, but not limited to, lack of social interactions, financial loss, and vaccination status may limit the generalizability of our findings. Third, sample bias was possible as patients with missing data were excluded. Fourth, there were no follow-up longitudinal data after discharge from the RTC. Thus, it was not possible to explore the associations between COVID-19–related acute signs and symptoms and the exacerbation of depression and anxiety, considering the acute and chronic periods in response to post–COVID-19 condition. Fifth, there may be a social desirability bias as patients self-reported their signs and symptoms and were not objectified by the caregivers. Lastly, while the mean quarantine period of participants was approximately 7 days, the PHQ-2 and GAD-2 measures depression and anxiety symptoms over the last 2 weeks. These timeline issues should be considered when interpreting the results. Further, other tools may be used in future studies.

### Conclusions

This study explored the association between acute signs and symptoms of COVID-19 and the exacerbation of depression and anxiety in patients with clinically mild COVID-19 who were admitted at RTCs. Among the acute signs and symptoms of COVID-19, exacerbation of depressive symptoms was associated with an abnormal SpO_2_ (OR 1.27, 95% CI 1.00-1.62) and sleep disorder (OR 0.60, 95% CI 0.36-0.98). Exacerbation of anxiety symptoms was associated with an abnormal BT (OR 1.08, 95% CI 1.01-1.16), sleep disorder (OR 1.1, 95% CI 1.06-1.14), and sore throat (OR 1.03, 95% CI 1.00-1.07). As anticipated, patients who had experienced initial psychological symptoms at admission were more likely to experience depression (OR 1.91, 95% CI 1.52-2.41) and anxiety (OR 1.08, 95% CI 1.01-1.16) than those who did not. Meanwhile, the PHQ-2 and GAD-2 scores measured at admission showed that lower the score, higher the possibility of exacerbation of both depression (OR 0.15, 95% CI 0.11-0.22) and anxiety (OR 0.13, 95% CI 0.10-0.19). Our results suggest the importance of considering further interventions that might mitigate the exacerbation of depression and anxiety in patients with an abnormal SpO_2_, abnormal BT, sore throat, and sleep disorders or those with initial psychological symptoms. In addition, we propose the use of the PHQ-2 and GAD-2, which are short and efficient scales, in the assessment of mental health for patients with clinically mild COVID-19 who are quarantined at home.

## Appendix

Multimedia Appendix 1 Detailed clinical characteristics by exacerbation of PHQ-2 and GAD-2 scores.

## Abbreviations

BT: body temperature
GAD-2: 2-item Generalized Anxiety Disorder
OR: odds ratio
PHQ-2: 2-item Patient Health Questionnaire
RPM: remote patient monitoring
RTC: residential treatment center
SNUH: Seoul National University Hospital
SpO_2_: saturation of percutaneous oxygen

## Data Availability

The data sets generated or analyzed during this study are not publicly available in accordance with the hospital’s regulations, adhering to the National Privacy Act and relevant guidelines. However, they may be available from the corresponding author on reasonable request after review and approval of the institutional review board and the institutional data steering committee.
