# Supplementary material for: The Association of Acute Signs and Symptoms of COVID-19 and Exacerbation of Depression and Anxiety in Patients With Clinically Mild COVID-19: Retrospective Observational Study
Source: JMIR Public Health Surveill. 2023 Jan 30;9:e43003. doi: 10.2196/43003 (PMC9926346; doi:10.2196/43003)
Supplement: Multimedia Appendix 1 [file publichealth_v9i1e43003_app1.docx]

Multimedia Appendix 1

Supplementary Table 1. Patients’ clinical characteristics by exacerbation of PHQ-2 scores.

| Clinical characteristics | Patients by exacerbation of PHQ-2 scores, No. (%) | |  |
| --- | --- | --- | --- |
|  | Not exacerbated (n=2,136) | Exacerbated (n=535) | *P* |
|  |  |  |  |
| **Demographic data** |  |  |  |
| Female | 925 (43.3%) | 317 (59.3%) | <.001 |
| Age under 30 | 658 (30.8%) | 90 (16.8%) | <.001 |
| Age 30 to 39 | 407 (19.1%) | 93 (17.4%) |  |
| Age 40 to 49 | 427 (20.0%) | 118 (22.1%) |  |
| Age 50 to 59 | 426 (19.9%) | 130 (24.3%) |  |
| Age over 60 | 218 (10.2%) | 104 (19.4%) |  |
| RTC A | 33 (1.5%) | 11 (2.1%) | .32 |
| RTC B | 239 (11.2%) | 75 (14.0%) |  |
| RTC C | 670 (31.4%) | 149 (27.9%) |  |
| RTC D | 1194 (55.9%) | 300 (56.1%) |  |
| Alpha variant | 559 (28.0%) | 147 (27.5%) | .001 |
| Delta variant | 1225 (57.4%) | 338 (63.2%) |  |
| Omicron variant | 312 (14.6%) | 50 (9.3%) |  |
| **Medical history** |  |  |  |
| Diabetes mellitus | 101 (4.7%) | 40 (7.5%) | .02 |
| Hypertension | 272 (12.7%) | 89 (16.6%) | .02 |
| Cardiovascular disease | 62 (2.9%) | 19 (3.6%) | .52 |
| Respiratory disease | 33 (1.5%) | 11 (2.1%) | .52 |
| Sleep disorder | 136 (6.4%) | 32 (6.0%) | .82 |
| Psychological treatment | 132 (6.2%) | 26 (4.9%) | .29 |
| **Initial psychological symptoms** | 977 (45.7%) | 296 (55.3%) | <.001 |
| **Initial PHQ-2 score** |  |  |  |
| 0 | 1261 (59.0%) | 380 (71.0%) | <.001 |
| 1 | 266 (12.5%) | 101 (18.9%) |  |
| Over 2 | 609 (28.5%) | 54 (10.1%) |  |
| **Smoking status** |  |  |  |
| Never smoked | 1265 (59.2%) | 335 (62.6%) | .04 |
| Ex-smoker | 447 (20.9%) | 118 (22.1%) |  |
| Smoker | 424 (19.9%) | 82 (15.3%) |  |
| **BMI** |  |  |  |
| Underweight/Normal | 942 (44.1%) | 240 (44.9%) | .53 |
| Overweight | 459 (21.5%) | 121 (22.6%) |  |
| Obese | 735 (34.4%) | 174 (32.5%) |  |
| **Abnormal vital signs** |  |  |  |
| SBP | 443 (20.7%) | 121 (22.6%) | .37 |
| DBP | 845 (39.6%) | 228 (42.6%) | .22 |
| HR | 66 (3.1%) | 20 (3.7%) | .53 |
| RR | 568 (26.6%) | 162 (30.3%) | .10 |
| BT | 283 (13.2%) | 92 (17.2%) | .02 |
| SpO_2_ | 45 (2.1%) | 27 (5.0%) | <.001 |
| **Acute respiratory COVID-19 related symptoms** |  |  |  |
| Cough | 1385 (64.8%) | 374 (69.9%) | .03 |
| Sputum | 1207 (56.5%) | 336 (62.8%) | .01 |
| Fever | 414 (19.4%) | 130 (24.3%) | .01 |
| Rhinorrhea | 1007 (47.1%) | 269 (50.3%) | .21 |
| Sore throat | 910 (42.6%) | 243 (45.4%) | .26 |
| Dyspnea | 182 (8.5%) | 68 (12.7%) | .004 |
| Chest pain | 315 (14.7%) | 106 (19.8%) | .005 |
| **Acute non-respiratory COVID-19 related symptoms** |  |  |  |
| Nausea | 143 (6.7%) | 64 (12.0%) | <.001 |
| Vomit | 58 (2.7%) | 25 (4.7%) | .03 |
| Abdominal discomfort | 330 (15.4%) | 123 (23.0%) | <.001 |
| Pain | 821 (38.4%) | 226 (42.2%) | .12 |
| Constipation | 320 (15.0%) | 106 (19.8%) | .01 |
| Diarrhea | 377 (17.6%) | 113 (21.1%) | .07 |
| Abdominal pain | 133 (6.1%) | 53 (9.8%) | .004 |
| Sleep disorder | 437 (20.5%) | 187 (35.0%) | <.001 |
| Loss of smell | 980 (45.9%) | 284 (53.1%) | .003 |
| Loss of taste | 963 (45.1%) | 280 (52.3%) | .003 |

Supplementary table 2. Patients’ clinical characteristics by exacerbation of GAD-2 scores.

| Clinical characteristics | Patients by exacerbation of GAD-2 scores, No. (%) | |  |
| --- | --- | --- | --- |
|  | Not exacerbated (n=2,148) | Exacerbated (n=523) | *P* |
|  |  |  |  |
| **Demographic data** |  |  |  |
| Female | 941 (43.8%) | 301(57.6%) | <.001 |
| Age under 30 | 656 (30.5%) | 92 (17.6%) | <.001 |
| Age 30 to 39 | 404 (18.8%) | 96 (18.4%) |  |
| Age 40 to 49 | 432 (20.1%) | 113 (21.6%) |  |
| Age 50 to 59 | 417 (19.4%) | 139 (26.6%) |  |
| Age over 60 | 239 (11.1%) | 83 (15.9%) |  |
| RTC A | 37 (1.7%) | 7 (1.3%) | .20 |
| RTC B | 245 (11.4%) | 69 (13.2%) |  |
| RTC C | 649 (30.2%) | 170 (32.5%) |  |
| RTC D | 1,217 (56.7%) | 277 (53.0%) |  |
| Alpha variant | 597 (27.8%) | 149 (28.5%) | .002 |
| Delta variant | 1,235 (57.5%) | 328 (62.7%) |  |
| Omicron variant | 316 (14.7%) | 46 (8.8%) |  |
| **Medical history** |  |  |  |
| Diabetes mellitus | 105 (4.9%) | 36 (6.9%) | .09 |
| Hypertension | 271 (12.6%) | 90 (17.2%) | .007 |
| Cardiovascular disease | 65 (3.0%) | 16 (3.1%) | 1.00 |
| Respiratory disease | 37 (1.7%) | 7 (1.3%) | .67 |
| Sleep disorder | 137 (6.4%) | 31 (5.9%) | .78 |
| Psychological treatment | 126 (5.9%) | 32 (6.1%) | .91 |
| **Initial psychological symptoms** | 1,005 (46.8%) | 268 (51.2%) | .08 |
| **Initial PHQ-2 score** |  |  |  |
| 0 | 1,111 (51.7%) | 350 (66.9%) | <.001 |
| 1 | 259 (12.1%) | 95 (18.2%) |  |
| Over 2 | 778 (36.2%) | 78 (14.9%) |  |
| **Smoking status** |  |  |  |
| Never smoked | 1,280 (59.6%) | 320 (61.2%) | .33 |
| Ex-smoker | 452 (21.0%) | 113 (21.6%) |  |
| Smoker | 416 (19.4%) | 90 (17.2%) |  |
| **BMI** |  |  |  |
| Underweight/Normal | 938 (43.7%) | 244 (46.7%) | .28 |
| Overweight | 472 (22.0%) | 108 (20.7%) |  |
| Obese | 738 (34.4%) | 171 (32.7%) |  |
| **Abnormal vital signs** |  |  |  |
| SBP | 455 (21.2%) | 109 (20.8%) | .91 |
| DBP | 848 (39.5%) | 225 (43.0%) | .15 |
| HR | 67 (3.1%) | 19 (3.6%) | .65 |
| RR | 575 (26.8%) | 155 (29.6%) | .21 |
| BT | 284 (13.2%) | 91 (17.4%) | .02 |
| SpO_2_ | 53 (2.5%) | 19 (3.6%) | .19 |
| **Acute respiratory COVID-19 related symptoms** |  |  |  |
| Cough | 1,411 (65.7%) | 348 (66.5%) | .75 |
| Sputum | 1,229 (57.2%) | 314 (60.0%) | .26 |
| Fever | 406 (18.9%) | 138 (26.4%) | <.001 |
| Rhinorrhea | 1,024 (47.7%) | 252 (48.2%) | .87 |
| Sore throat | 911 (42.4%) | 242 (46.3%) | .12 |
| Dyspnea | 185 (8.6%) | 65 (12.4%) | .009 |
| Chest pain | 315 (14.7%) | 106 (20.3%) | .002 |
| **Acute non-respiratory COVID-19 related symptoms** |  |  |  |
| Nausea | 143 (6.7%) | 64 (12.2%) | <.001 |
| Vomit | 60 (2.8%) | 23 (4.4%) | .08 |
| Abdominal discomfort | 339 (15.8%) | 114 (21.8%) | .001 |
| Pain | 822 (38.3%) | 225 (43.0%) | .052 |
| Constipation | 325 (15.1%) | 101 (19.3%) | .02 |
| Diarrhea | 379 (17.6%) | 111 (21.2%) | .07 |
| Abdominal pain | 129 (6.0%) | 57 (10.9%) | <.001 |
| Sleep disorder | 445 (20.7%) | 179 (34.2%) | <.001 |
| Loss of smell | 982 (45.7%) | 282 (53.9%) | .001 |
| Loss of taste | 963 (44.8%) | 280 (53.5%) | <.001 |
